# Effect of the Formation Rate on the Stability of Anode-Free Lithium Metal Batteries

**DOI:** 10.1021/acsenergylett.4c02258

**Published:** 2024-09-06

**Authors:** Soochan Kim, Pravin N. Didwal, Juliane Fiates, James A. Dawson, Robert S. Weatherup, Michael De Volder

**Affiliations:** † Department of Engineering, University of Cambridge, Cambridge CB3 0FS, United Kingdom; ‡ School of Chemical Engineering, Sungkyunkwan University, Suwon 16419, Republic of Korea; ¶ Department of Materials, University of Oxford, Oxford OX1 3PH, United Kingdom; § The Faraday Institution, Quad One, Harwell Science and Innovation Campus, Didcot OX11 0RA, United Kingdom; ∥ Chemistry - School of Natural and Environmental Science, Newcastle University, Newcastle upon Tyne NE1 7RU, United Kingdom

## Abstract

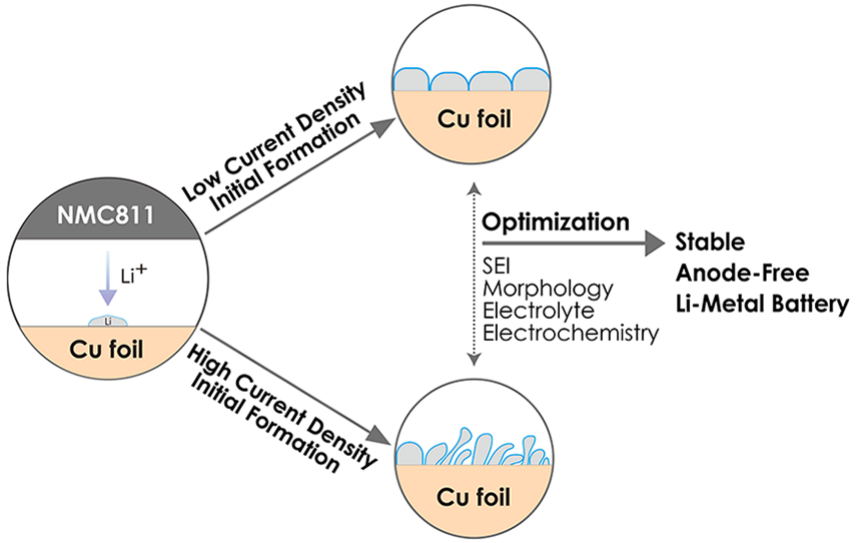

Anode-free Li-ion batteries (AFBs), where a Cu current collector is used to plate and strip Li instead of a classic anode, are promising technologies to increase the energy density of batteries. In addition, AFBs are safer and easier to manufacture than competing Li-metal anodes and solid-state batteries. However, the loss of Li inventory that occurs during the operation of AFBs limits their lifespan and practical application. In this study, we find that, in particular, the current density used during the formation of AFBs has a considerable impact on the cycling stability of the cell. We optimize the formation protocol based on experimental and computational observations of thresholds associated with morphological changes in the plated Li and the chemical composition of the solid–electrolyte interphase. Unlike graphite anodes, which require slow formation cycles, AFBs exhibit improved cycling behavior when formed at the highest current densities that avoid dendritic Li formation. We verify that this strategy for optimizing the formation current density is effective for three different electrolyte formulations and, therefore, provides a straightforward universal rationale to optimize the formation protocols for AFBs.

The idea of using Li-metal as a battery anode dates back to Whittingham’s studies in the early 1970s and is still attractive to date because of lithium’s high specific capacity (3861 mAh/g), low redox potential (−3.04 V vs standard hydrogen electrode), and low density (0.534 g/cm^3^). Li-metal anodes are therefore an interesting contender to achieve batteries that go beyond conventional Li-ion batteries (LiBs) with an energy density of 300 Wh/kg.^1−3^ However, the processing of metallic Li requires a controlled air- and moisture-free environment, and the Li anode should ideally be no more than 20 μm thick to achieve high energy densities.^1,4,5^ This adds further to the manufacturing complexity and battery costs.^1,4,5^ These issues have led to the development of so-called zero-excess, anode-less, or anode-free Li-metal batteries (AFBs).

AFBs rely on the fact that standard cathodes contain all the Li needed for battery operation, and a Cu foil anode current collector is therefore, in principle, sufficient to plate Li on during the charging process and strip it during discharge. While in theory this battery technology is very attractive from both manufacturing and energy density perspectives, in practice these cells suffer from very fast capacity fade as Li inventory is lost through side reactions during cycling and they lack the reservoir of excess Li from which Li-metal anodes benefit. To improve the stability of AFBs, new current collector designs, electrolytes, and cycling protocols and increases in stack pressure have been proposed, with remarkable improvements in Li plating/stripping behavior.^4,6−8^ AFBs with new electrolytes, such as dual salt electrolytes (DSEs) and localized high-concentration electrolytes (LHCEs), show stable cycling behaviors, with a high capacity retention of over 80% after 200 cycles.^6,9,10^ However, these electrolyte systems and other solutions proposed for AFBs are typically expensive and require unique operation environments to achieve improved battery performance (e.g., high temperature, high stack pressure, narrow potential ranges). Meanwhile, new cycling protocols have been proposed as a cost-effective way to improve the stability of Li-metal anodes and AFBs. For instance, using asymmetric cycling with fast stripping rates is reported to favor stripping from the tip of Li deposits and therefore reduce the formation of dead Li.^4,11−13^ While these new cycling protocols provide improved battery performance, the most important cycle in the AFBs would be the early stages, in which the initial anode is formed. The initial formation process at certain temperatures (hot or cold) has contributed to the improvement of the subsequent cycling behavior of AFBs,^4,6,11^ but there is still a lack of understanding regarding the optimal cycling conditions. Furthermore, despite the importance of the initial formation cycle, most AFB studies seem to adopt either slow C/10 or slow formation protocols optimized for graphite rather than for AFB anodes.

In this study, we investigate the impacts of current density (CD) during the initial formation cycle on the subsequent cycling stability of AFBs and optimize the anode-formation protocol. Changes in the charging CD not only result in the formation of Li-metal with different structures and solid–electrolyte interphase (SEI) compositions on the current collector but also affect the stripping behaviors of Li-metal during discharging. As a result, we found that this optimal formation CD varies with the electrolyte composition, which confirms the need for an electrolyte-tailored formation protocol distinct from the conventional slow formations used for graphite anodes. For AFBs, the initial formation at the highest current density that still avoids dendritic Li plating is found to be advantageous.

AFBs were constructed from a commercial LiNi_0.8_Mn_0.1_Co_0.1_O_2_ (NMC 811) cathode with an areal capacity of ∼3 mAh/cm^2^, a Cu foil, a polyolefin separator (Celgard 2325), and a commercial electrolyte of 1 M LiPF_6_ in ethylene carbonate/diethyl carbonate (EC/DEC, 1/1, v/v), as shown in Figure 1a. Although this standard electrolyte is not formulated for a long cycle life, it is used as a reference to study the effect of the CD at the formation cycle on degradation. The initial charging CD was changed from C/20 to 4C, and the discharging CD was fixed at C/2. After the initial formation cycle, all batteries were cycled using a typical AFB protocol (charging at C/5 and discharging at C/2). As shown in Figure 1b, increasing the formation CD increases the cell voltage overpotential as expected, but at 2C and 4C abnormal voltage changes reaching over 4.3 V (vs Li/Li^+^, general cutoff voltage) were observed early in the cycle. At a CD of 4C, the potential reached beyond 4.7 V, where significant electrolyte decomposition is expected. Therefore, to ensure reliable results, we set the initial charging CD to the range from C/20 to 1C and conducted a battery cycling test with prepared AFBs (Figure S1).

**Figure 1:**
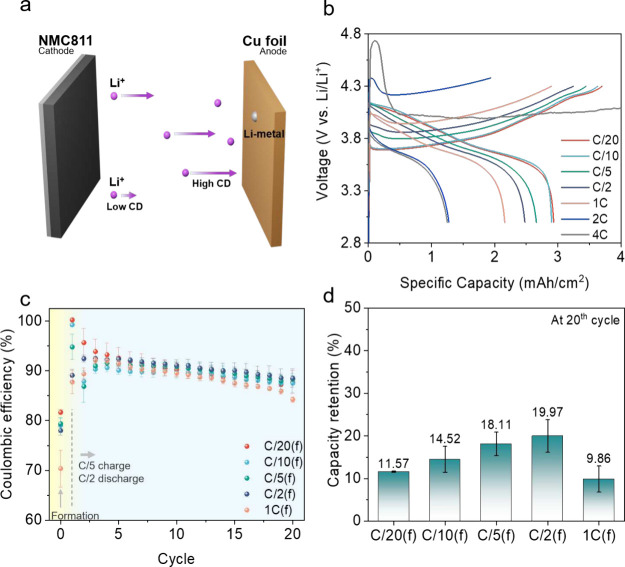
(a) Schematic of a Cu∥NMC811 AFB system. (b) Initial cycle voltage profiles of Cu∥NMC811 AFBs with various initial charging CDs and a fixed discharging CD at C/2. (c) Coulombic efficiency of AFBs (detailed cycling results in Figure S1). (d) Capacity retention after the 20th cycle of AFBs.

Figure 1c shows the Coulombic efficiency (CE) of the anode-free cells for 20 cycles (after formation). The CE of the initial formation cycle is seen to decrease as the charging CD increases (detailed in Table S1). The initial low CE value is attributable to at least two factors associated with the Li plating morphology in the first cycle: (i) faster formation CDs lead to smaller Li deposits and hence a higher surface area for SEI formation; (ii) after the first discharge, the stripped Li and SEI template subsequent Li plating morphology and therefore influence the CE and lifetime. A more detailed discussion on the post-mortem analysis of Li deposits is provided further below. Interestingly, increasing the formation CD increases the cycling stability, with the most stable cycling behavior observed at a formation CD of C/2 achieving capacity retentions of 19.97 ± 3.82% compared to 11.57 ± 0.16% for standard slow C/20 formation protocols (Figure 1d). These observations motivate the need for reconsidering the current density at which the initial formation of AFBs is carried out.

To understand how the formation cycle affects the battery lifetime, electrochemical, structural, and chemical characterizations were performed. First, electrochemical impedance spectroscopy (EIS) was carried out to understand changes in impedance in the cell; however, as shown in Figure 2a,b, the semicircles from the Nyquist plots tend to overlap, making it challenging to separate the evolution of different effects, necessitating an alternative method to analyze our EIS data, such as bode plots or distribution of relaxation times (DRT)-EIS.^14,15^ The latter was adopted here, because based on previous studies,^14,16,17^ three relaxation times (τ_1_, τ_2_, and τ_3_) can be extracted and assigned to cathode charge transfer, anode charge transfer, and the Li-ion transport in the SEI layer (anode), respectively (Figures 2b and S2). Among them, we focused on the changes in τ_3_ to investigate the changes in the SEI layer: a rapid increase in the τ_3_ peak between the state of charge (SoC) 20% and SoC 100% was observed with a charging CD of C/2 or higher. The values plotted in Figure 2c are not absolute, as they depend on experimental factors such as electrolyte wetting and absorption rate; therefore, we study the ratio of values (Δτ_3_ = γ(τ) at SoC 100% – γ(τ) at SoC 20%) shown in Figure S2f. Accordingly, the huge change in τ_3_ values between SoC 20% and 100% at high CD meant that the transport of Li-ions in the SEI layer was delayed. However, the reason for the delayed Li-ion transport can be explained in various ways depending on the change in the SEI composition, thickness, structure, and so on. Therefore, we investigated which factors caused this change in SEI, considering various factors. First, we performed classical molecular dynamics (MD) simulations of electrolyte components (1 M LiPF_6_ in EC/DEC) at the Cu surface. These simulations do not capture the reactions that form the SEI but shed light on the processes taking place at the beginning of the initial formation cycle.^18−25^ To correctly describe the electrode–electrolyte interaction process, we employed the constant potential method to allow for fluctuations in the charge of the electrode according to its interaction with the electrolyte during the equilibration stage, as shown in Figure S3. Previous studies^18,19^ indicate that charge modes based on applied current closely correspond to those based on applied voltage. This suggests that varying CDs can be computationally modeled by using different voltage potentials. Figure S4 illustrates the impact of voltage on the electrolyte environment at the interface, with voltages ranging from 0.1 to 2 V representing an increase in the CD from C/20 to 1C. For both solvents (EC and DEC), nonsignificant changes are observed with the increase of electrode voltage. In contrast, for Li^+^, we see an increase in the concentration at the interface. The formation of multiple concentration peaks within the first ∼4 Å and new peaks in the region 5–12 Å from the surface is observed as the voltage is increased (Figure S4a–c). Furthermore, the Li^+^ solvation close to the current collector is expected to influence SEI formation and Li plating.^22^ Considering this, we evaluate the RDF (*g*(*r*)) of Li–O_EC_, Li–O_DEC_, and Li–P_PF_6__ at the inner layer within 5 Å from the surface, the oxygen atoms from EC and DEC used in this analysis are represented in Figure S4d.

**Figure 2:**
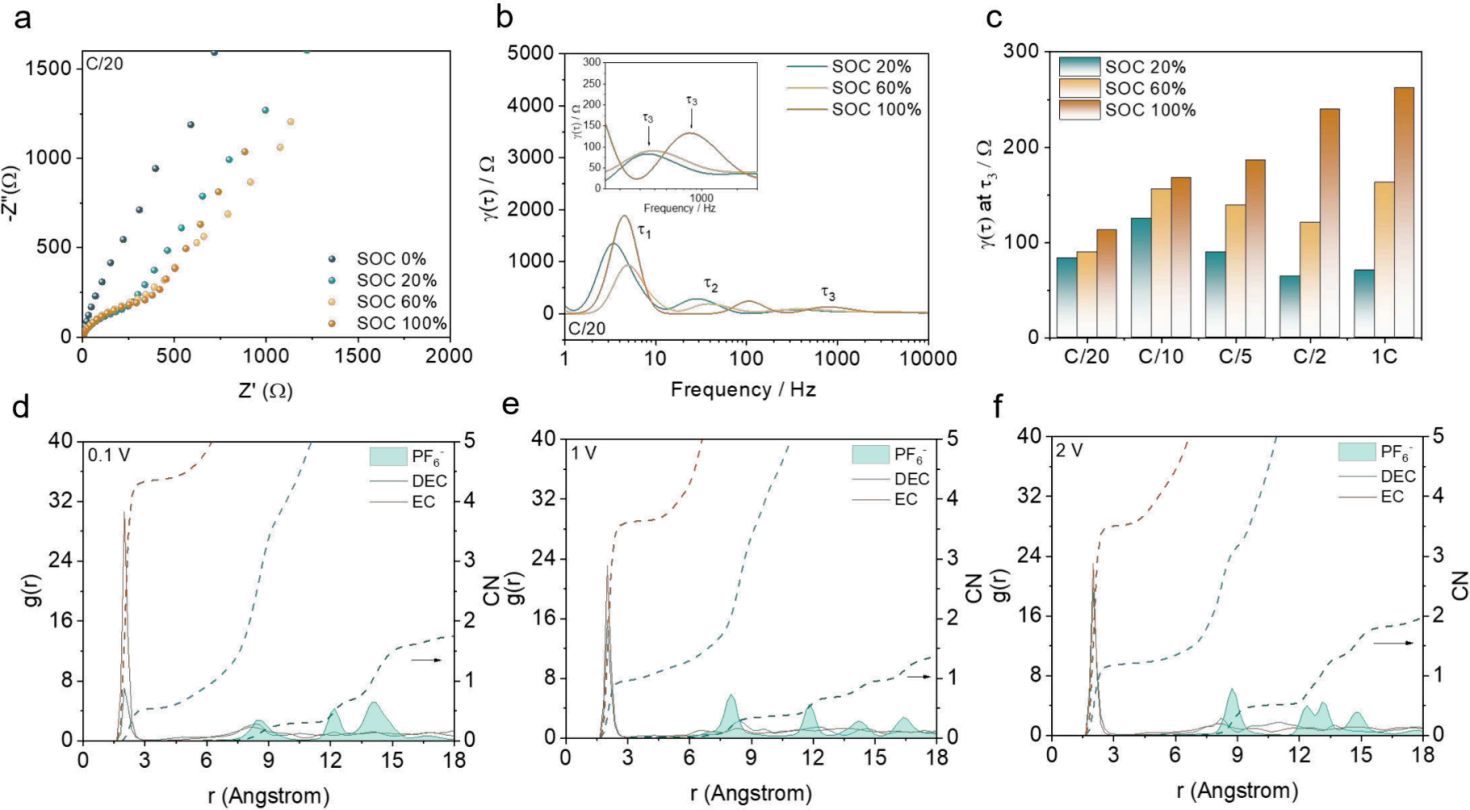
(a, b) Nyquist plots and DRT graphs calculated from EIS measurements at different SoC values at C/20. (c) γ(τ) values at τ_3_ obtained according to the SoC and initial charging CD. (d–f) Molecular dynamics (MD) simulations of Li^+^-solvation structure in 1 M LiPF_6_ in EC/DEC electrolyte in contact with the Cu (111) surface: radial distribution function (RDF, *g*(*r*)) and coordination number (CN) plots at 0.1, 1, and 2 V in the innermost layer of 5 Å.

Figure 2d–f presents the *g*(*r*) values for the applied voltages. The *g*(*r*)’s from Li–O_EC_ and Li–O_DEC_ exhibit a single peak at 2 Å for the interaction between Li^+^–EC and Li^+^–DEC pairs at all voltages.^26^ As the voltage increases, the solvation between Li–O_EC_ decreases, while DEC begins to participate more effectively in the solvation of Li^+^. The flexibility of DEC compared to the EC ring can lead to competition for Li^+^ solvation. For PF_6_^–^, a peak is observed around 8 Å, which increases in height with voltage, indicating that PF_6_^–^ is more frequently found at higher voltages. In addition, a denser secondary peak forms at 13 Å at 2 V, evidencing a more crowded environment in the vicinity of Li^+^ at a higher voltage. Regarding the coordination number (CN), the increase in voltage results in an overall enhancement in the solvation of Li^+^ by DEC and PF_6_^–^, with a decrease in the presence of EC, as shown in Table S2. These solvation numbers can be explained by the abilities of EC, DEC, and PF_6_^–^ to solvate Li^+^. The donor number (DN) measures the abilities of solvents and anions to donate lone pairs of electrons to coordinate with accepting ions in the electrolyte. The DN values of EC (∼17 kcal mol^–1^), DEC (∼15 kcal mol^–1^), and PF_6_^–^ (∼2.5 kcal mol^–1^)^27^ help explain the observed order of preference in Li^+^ solvation: EC > DEC > PF_6_^–^, as shown by the calculated coordination numbers (Table S2). Furthermore, even though EC is more likely to solvate Li^+^, the electric field contributes to a decrease in the amount of EC in the Li^+^ solvation shell, leading to its replacement by DEC and PF_6_^–^. This highlights the contribution of operational conditions in the formation cycle to the modulation of electrolyte organization near the Cu interface.

Considering the mechanism proposed by Lan et al.^27^ and Aurbach et al.,^28^ PF_6_^–^ needs to be surrounded by Li^+^ to support the formation of LiF. The presence of an anion in the first Li^+^ solvation shell or the promotion of a stronger Li^+^–PF_6_^–^ interaction leads to an inorganic-rich SEI. Moreover, previous literature^29^ suggests that the combined presence of DEC and EC in Li^+^ solvation close to the interface leads to a more stable SEI. Our simulation results show that the increase of the electric field during the formation cycle contributes to the Li^+^–PF_6_^–^ interaction and promotes a higher presence of DEC in the Li^+^ solvation shell. Additionally, the calculation of *g*(*r*) and CN in the middle of the simulated cell (25–30 Å), presented in Figure S4, shows that the solvation is unaffected by the electric field in this area. This underscores the importance of operational conditions in the formation cycle at the interface.

Second, we investigated the composition of the SEI, which shows changes with the formation protocol, through the X-ray photoelectron spectroscopy (XPS) measurements shown in Figures 3a and S5 (peaks were assigned by considering the literature^30−32^). As the charging CD increases, the intensities of the organic and inorganic-based components such as LiF, Li_2_CO_3_, LiOH, and Li alkyl carbonates are seen to increase. In particular, it can be seen that the change in the inorganic SEI is large. An inorganic-rich SEI on Li-metal anodes is known to have unique advantages in dendrite suppression owing to its improved mechanical properties and the blocking of electron tunneling.^20,21,33^ These results are also consistent with the simulation results in Figure 2d–f. Post-mortem scanning electron microscopy (SEM) imaging after the first formation charge shows that the plated Li has a smooth morphology and large grains at a low-charging CD; moreover, as the CD increased, the size of the Li grains decreased (detailed in Figure S6), with a transition from plate to a fibrous dendrite growth at 1C. These observed changes in morphology help explain the changes in CE in Figure 2 discussed above. However, to confirm whether the grain size of the initially plated Li is a critical factor that can lead to stable battery cycling, changes after discharging must also be observed.

**Figure 3:**
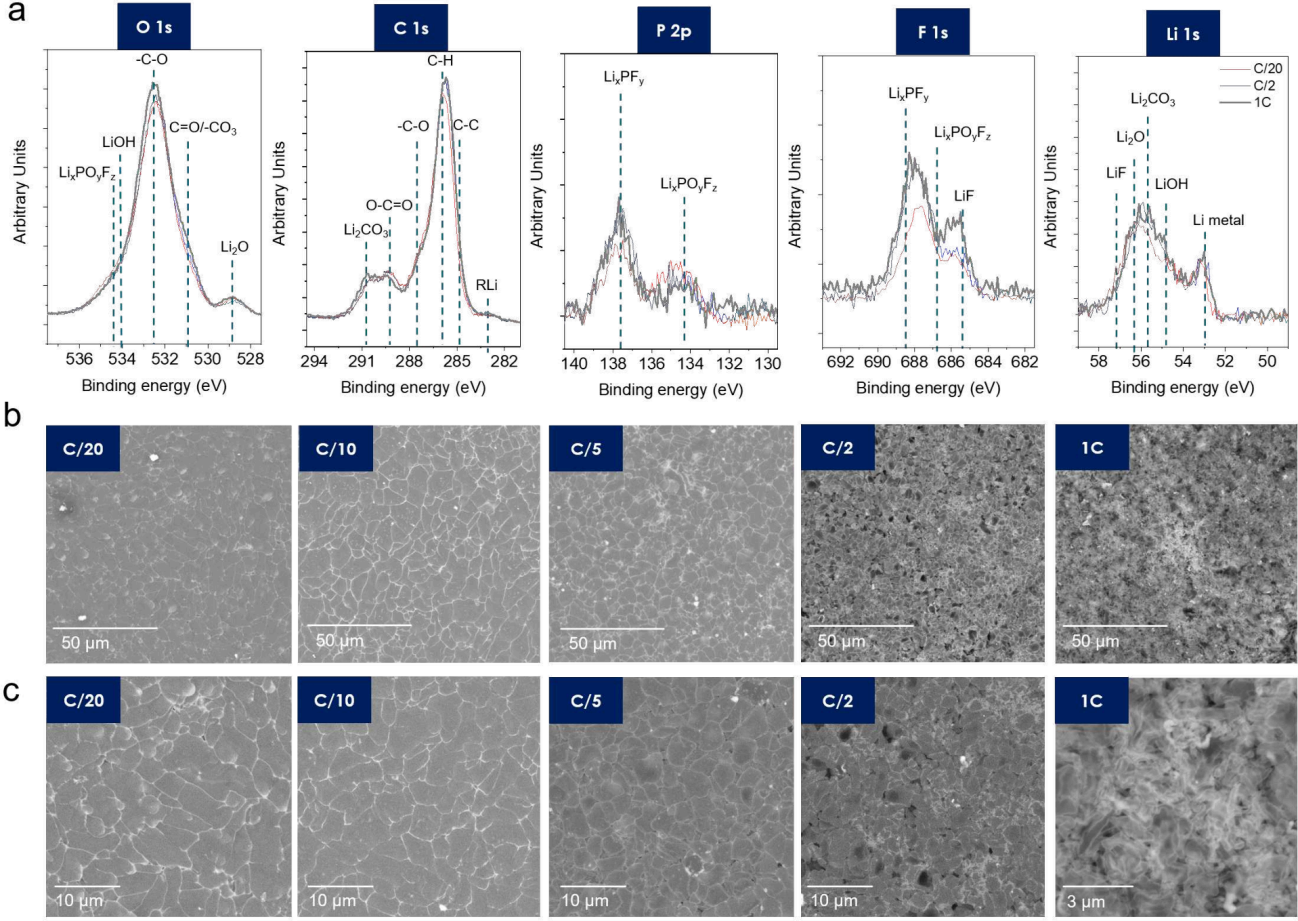
(a) XPS of the anodes after the initial charging at C/20, C/2, and 1C. (b) SEM images of the anodes after the initial charging and (c) magnified images of panel b.

The anode surface was investigated after the initial discharge, during which Li was stripped from the anode and recovered in the cathode. Li plating and stripping are not fully reversible; therefore, after discharge, the anode is covered in residual Li and SEI, as shown in Figure 4a. At low CDs, Li was stripped entirely from certain areas of the current collector, resulting in exposed Cu foil (bright areas in Figure 4b). Meanwhile, the cells with an initial charging CD of C/5 or faster showed densely packed nanomorphologies covering the entire anode after stripping.

**Figure 4:**
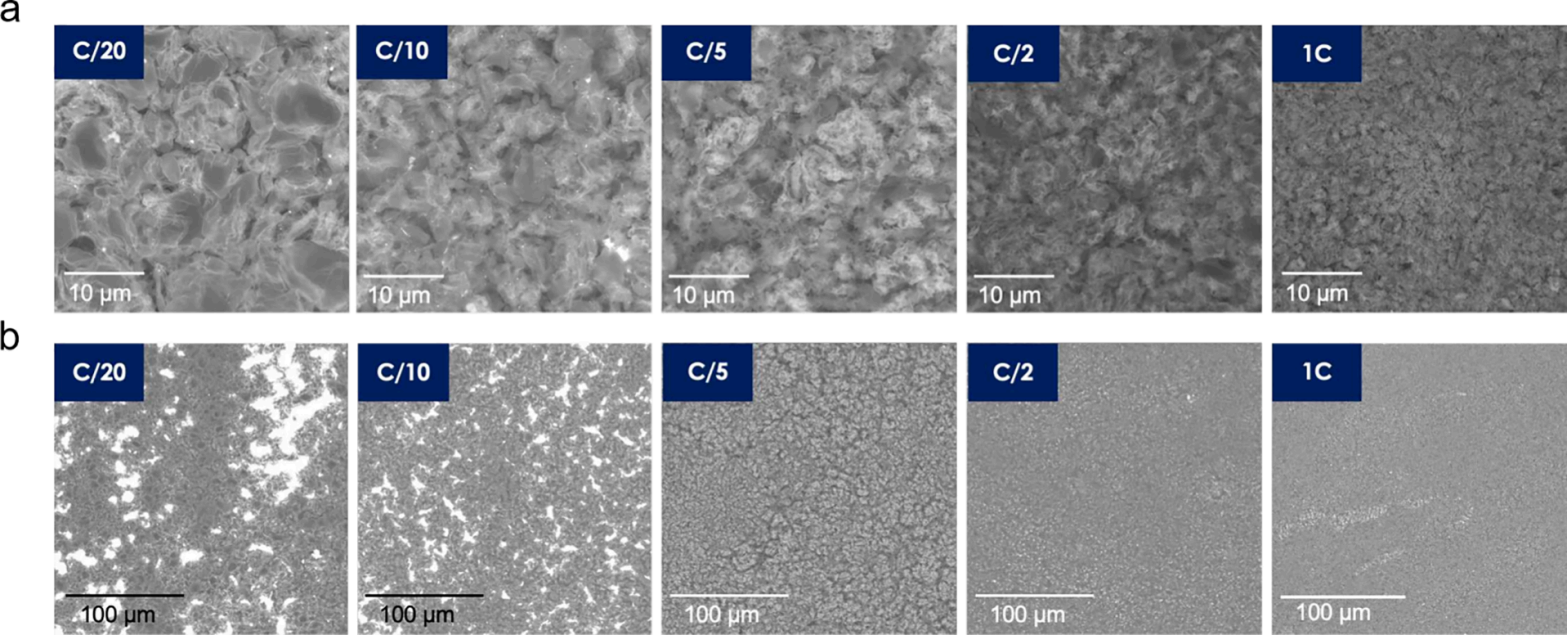
(a) SEM images of the anodes after the initial discharging and (b) low-magnified images of panel a.

Figure 5 demonstrates the relationship between the initial charging CD and the structural behaviors of Li-metal anodes considering our results and reported literature.^34−36^ As schematically depicted, during the initial formation process at slow-charging CDs, large Li flakes are deposited on the current collector, and when these Li structures are stripped, an exposed Cu surface is created. In subsequent cycles, additional energy barriers must be overcome to plate Li on exposed Cu compared to the residual Li/SEI region, which may lead to accelerated Li deposition on Li residues from previous cycles, leading to an uneven Li morphology and additional SEI formation.^37−40^ In contrast, the cells with a high initial charging CD leave a dense SEI layer behind, with no exposed Cu. We suspect that this layer may lead to a denser Li deposition in subsequent cycles. However, above a certain limit of initial charging CD, dendritic Li is formed, which accelerates the degradation of AFBs. This is in agreement with the battery cycling results shown in Figure 1 and cross-sectional SEM images (Li-metal anode after second charging) in Figure S7.

**Figure 5:**
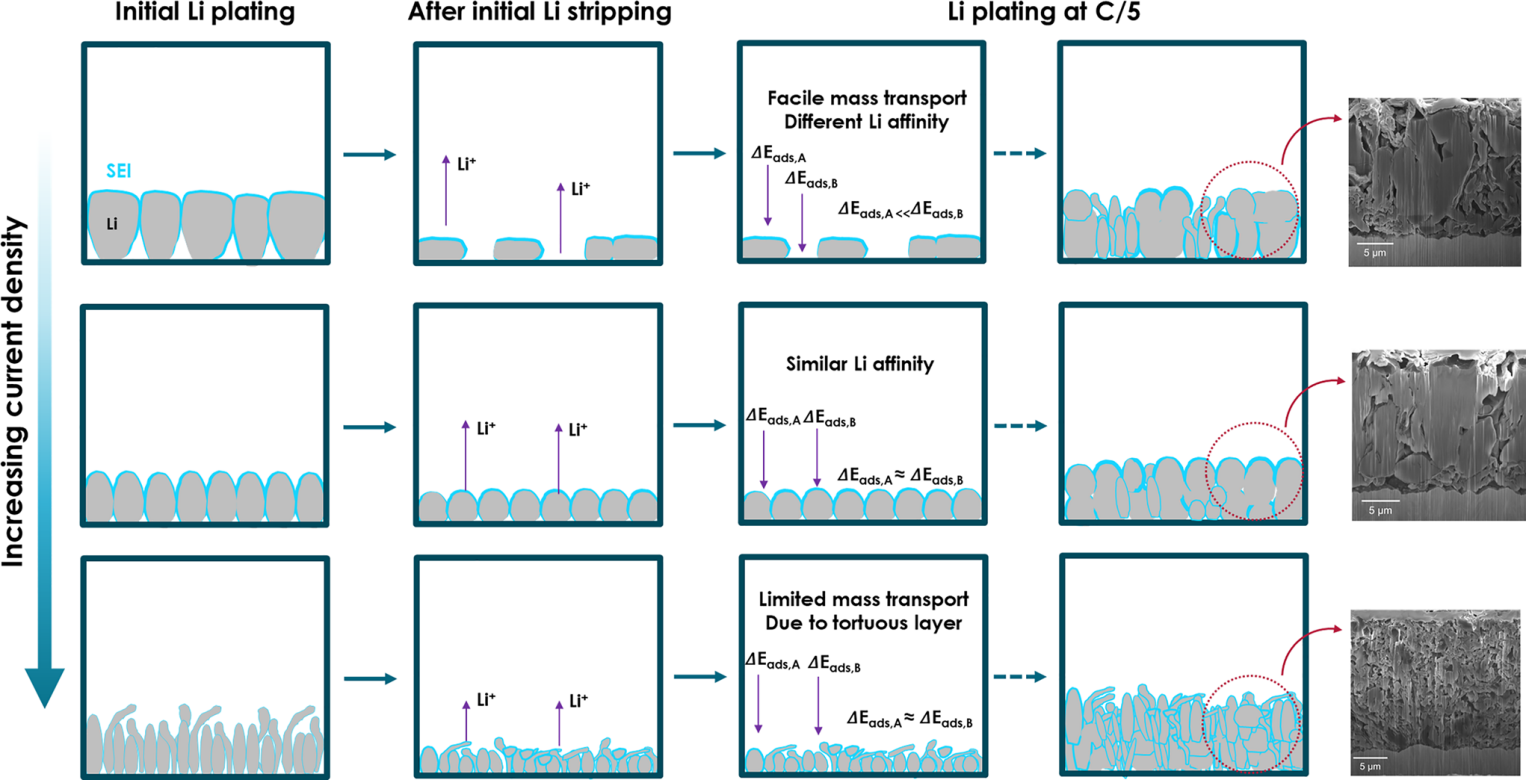
Schematic of the relationship between the initial charging CD and the characteristics of the Li-metal anode.

Next, we investigated whether the occurrence of an optimal formation CD at the maximum CD before dendrites are formed is universally applicable to different types of electrolytes. We selected two alternative electrolyte formulations that are popular in recent literature on AFBs, namely one based on dual-salts electrolyte (DSE, 0.6 M lithium difluoro(oxalato)borate + 0.6 M lithium tetrafluoroborate in 4-fluoro-1,3-dioxolan-2-one/diethyl carbonate (1/2, v/v)) and one based on the localized high-concentration electrolyte (LHCE, lithium bis(fluorosulfonyl)amide/1,2-dimethoxyethane/1,1,2,2-tetrafluoroethyl-2,2,3,3-tetrafluoropropyl ether, 1/1.2/3 (molar ratio)).^6,10^Figure 6 shows the cycling results for AFBs with these new electrolytes using the above cycling protocol. As shown in Figure S8, after the initial charging, these AFB anodes show trends similar to the ones above, with a decrease in the size of Li grains with increasing CD. Consistent with previous results, the cells that had the highest CD during formation that did not lead to dendrite formation achieved the best cycling performance. However, depending on the electrolyte formulation, this transition happens at different CDs, as expected from their differences in ionic conductivity and SEI composition. In the case of DSEs, the optimum formation in our test conditions was C/5, and in the case of LHCEs, it was C/10.

**Figure 6:**
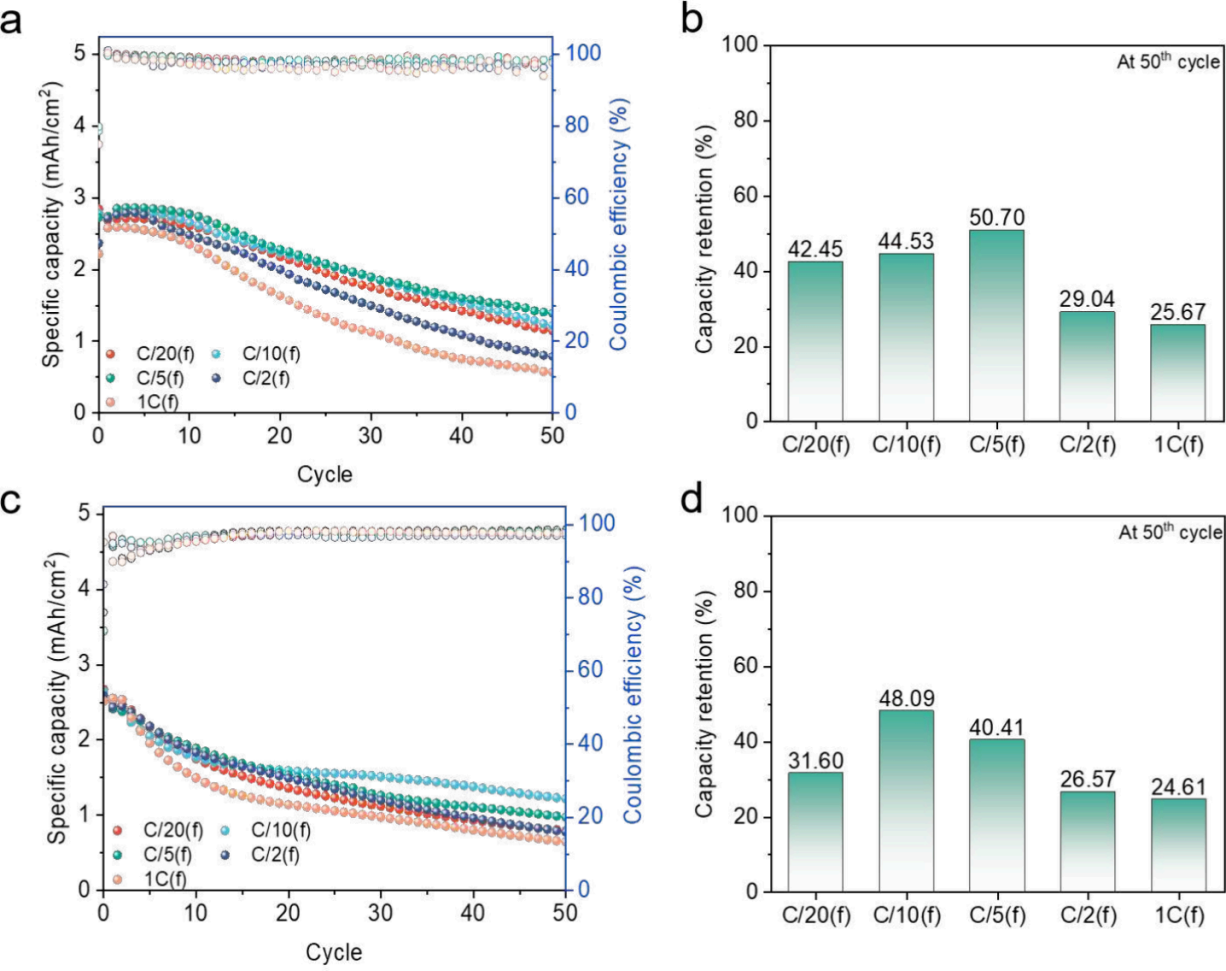
Cycling performance and capacity retention of cells after initial formation (a, b) with the DSE and (c, d) with the LHCE.

In summary, we have demonstrated that the initial formation protocol has a strong influence on the subsequent cycling stability of AFBs. Depending on the initial charging CD, Li-metal with different morphologies and SEIs with different chemical compositions were formed, which affect the Li plating and stripping during cycling. Unlike classic anodes, which are best formed very slowly, we found for three different classes of electrolytes that the most stable cycling behavior was observed for cells formed at the highest CD before Li dendrites were developed. This CD is a unique property of the specific electrolyte used, and the rationale developed herein offers a powerful tool to identify and optimize the formation conditions.

## Supporting Information Available

Experimental section, detailed battery cycling behaviors, detailed DRT graphs, detailed MD simulations, XPS spectra of anodes after initial charging, Li grain size calculated by ImageJ software, SEM images of anodes after initial charging (AFBs with the DSE and the LHCE), cross-sectional SEM images, and detailed CE of AFBs (PDF)
