# Supplementary material for: Effect of the Formation Rate on the Stability of Anode-Free Lithium Metal Batteries
Source: ACS Energy Lett. 2024 Sep 6;9(10):4753–60. doi: 10.1021/acsenergylett.4c02258 (PMC11474951; doi:10.1021/acsenergylett.4c02258)
Supplement: Supplementary file 1 — nz4c02258_si_001.pdf [file nz4c02258_si_001.pdf]

Supporting Information

Effect of the Formation Rate on the Stability of Anode-Free Lithium Metal Batteries

Soochan Kim[1,2,], Pravin N. Didwal[3,4], Juliane Fiates[4,5], James A. Dawson[4,5], Robert S. Weatherup[3,4], and Michael De Volder[1,4,*]*

[1]Department of Engineering, University of Cambridge, Cambridge CB3 0FS, United Kingdom
[2]School of Chemical Engineering, Sungkyunkwan University, Suwon 16419, Republic of Korea
[3]Department of Materials, University of Oxford, Oxford OX1 3PH, United Kingdom
[4]The Faraday Institution, Quad One, Harwell Science and Innovation Campus, Didcot OX11 0RA, United Kingdom
[5]Chemistry – School of Natural and Environmental Science, Newcastle University, Newcastle upon Tyne NE1 7RU, United Kingdom

1. Experimental

1.1. Electrolyte Preparation

First, 1M $LiPF_6$ in an ethylene carbonate/diethyl carbonate (1/1, *v*/*v*) solution (battery grade) was purchased from Sigma-Aldrich. The dual salt electrolytes (DSEs) and localized high-concentration electrolytes (LHCEs) were mixed from pure chemicals in an argon-filled glove box (MBRAUN, Germany), in which both the moisture and oxygen contents were controlled to below <0.5 ppm. The chemicals used include 4-fluoro-1,3-dioxolan-2-one (FEC, Flourochem), diethyl carbonate (DEC, anhydrous, Sigma-Aldrich), lithium difluoro(oxalato)borate (LiDFOB, Sigma-Aldrich), lithium tetrafluoroborate ($LiBF_4$, Sigma-Aldrich), lithium bis(fluorosulfonyl)amide (LiFSI, Fluorochem), 1,2-dimethoxyethane (DME, anhydrous, Sigma-Aldrich), and 1,1,2,2-tetrafluoroethyl-2,2,3,3-tetrafluoropropyl ether (TTE, TCI). The DSE was prepared by mixing 0.6 M LiDFOB and 0.6 M $LiBF_4$ in FEC/DEC (1/2, v/v), and the LHCE was composed of LiFSI-1.2DME-3TTE (molar ratio) [1, 2].

1.2. Characterization

Scanning electron microscopy (SEM) morphology observations of the samples were conducted on a Phenom Pro SEM (Thermo Fisher, U.S.A.) at 10.0 kV in the backscattering mode. To prepare the samples for SEM and XPS, the cells were disassembled in an argon-filled glove box, rinsed three times with dimethyl carbonate (DMC, anhydrous, Sigma-Aldrich) to remove the residual salt, and dried. Dried electrodes were transferred to the SEM chamber using inert atmosphere transfer vessels to minimize the air-exposure. The Li grain areas were estimated using ImageJ software [3]. To investigate the cross-sectional morphology of Li-metal anodes, cryo-focused ion beam (FIB) milling was performed using the Helios 5 UX (Thermo Fisher, U.S.A.). The samples were stored under Ar-filled glovebox and then they were

transferred to the microscope using an airless transfer vessel to the FIB milling machine. Li metal anodes were milled at cryogenic temperatures (−160 °C) using a cryogenic stage. For X-ray Photoelectron Spectroscopy (XPS) analysis, the anodes were transferred to the XPS measurement chamber using inert atmosphere transfer vessels to eliminate air exposure. XPS spectra were collected using a Phi XPS Versaprobe III with an Al Kα X-ray source at 1.486 keV (probe depth < 10 nm), under high vacuum conditions (>10^{-6} mbar). All XPS spectra were analyzed using CASA-XPS software with Shirley background subtraction.

1.3. Electrochemical characterization

Electrochemical characterization was performed using CR2032 coin-type cells (Cambridge Energy Solutions Ltd., U.K.). Battery cells were assembled with a single-crystal $LiNi_{0.8}Mn_{0.1}Co_{0.1}O_2$ (NMC 811) electrode as the cathode (13 mm diameter, the NMC811 powder was purchased from Targray and provided to LiFun Technology, China, the single-side coated on the Al foil with the active materials are 15.0 mg/cm^2 (96.4% active material)), a copper foil as the anode current collector (15 mm diameter, 8 μm thickness, MTI Corp. U.S.A.), Celgard 2325 as the separator, one spacer (1 mm thickness), and electrolytes (50 μL) in the argon-filled glove box. Cell testing was performed via galvanostatic charge-discharge on a BCS 805 series battery cycler (BioLogic, France), stack pressure of the cells was below 1MPa, the cells were placed in an environmental chamber at 26 °C. The battery testing was performed with five samples and the error bars were obtained on three samples, excluding the minimum and maximum result values. During the first charge, Li metal was directly transferred from the cathode plates to the copper current collector. The cycling protocol comprised an initial formation cycle (charged to 4.3 V vs. Li/Li^+ at various current densities (CDs)and discharging to 3 V at C/2) and a testing cycle (charged to 4.3 V at C/5 and discharging to 3 V

at C/2). 1C corresponds to a CD of 200 mA/g based on the NMC 811 material. Electrochemical impedance spectroscopy (EIS) measurements were performed using a VMP-3 (BioLogic, France) in a frequency range from 1 MHz to 10 mHz with a perturbation amplitude of ±10 mV. Distribution of relaxation times (DRT) profiles were computed from the experimental EIS data using the DRTtools software. [4]

1.4. Simulation Details

We performed classical molecular dynamics simulation using LAMMPS (Large-scale Atomic/Molecular Massively Parallel Simulator).[5] The force field parameters for the DEC and EC were obtained from the OPLS-AA (Optimized Potentials for Liquid Simulations All Atom) [6,7] using the LigParGen webserver.[8] The charges were reassigned using CHelpG at B3LYP/def2-TZVPP level as implemented in ORCA, version 5.0.[9] For PF_6^- and Li^+ the force field parameters were taken from Lopes et al. [10] and Jensen et al. [11] with a charge scaling of 0.8. The initial configurations were prepared by randomly assembling 338 molecules of EC, 186 of DEC, and 47 of $LiPF_6$ in the simulation box using Packmol [12] and Moltemplate [13]. For the bulk simulations, an energy minimization using the steepest descent algorithm was performed. Followed by a 3 ns equilibration stage in the isothermal-isobaric ensemble (NPT), and a 31 ns production stage in the canonical ensemble (NVT), the first ns was discarded due to equilibration purposes. A Nosé–Hoover thermostat [14,15] and Parrinello-Rahman barostat [16] were used, and the electrostatic interactions were computed using the particle–particle particle–mesh scheme (PPPM) [17], applying a cutoff of 12 Å. Two temperatures were simulated 25 and 35 °C. For the interface, the simulations were performed using the NVT ensemble, and periodic boundary conditions were applied in the x and y directions. The Yeh–Berkowitz condition was applied to prevent unwanted slab-slab interactions. [18,19] A vacuum

space of 120 Å was added to avoid spurious interaction in the z-direction. The electrode surface was composed of four layers of Cu (111) with 224 atoms each. The dimensions were 34.51 x 33.21 Å in the x and y directions, separated apart by 60 Å of electrolyte. The Lennard-Jones parameters for Cu were taken from Heinz et al. [20] An equilibrium simulation was conducted using zero constant charge for 30 ns. A second step of equilibration using the constant potential method (CPM) [21-24] was performed for 4 ns, followed by production runs of 2 ns. Multiple trajectory analyses were applied in which 6 blocks were used to calculate the results in the production stage. Three potential differences were used 0.1, 1, and 2 V. The electrolyte-Cu interface simulations were conducted at a temperature of 26 °C.

2. Figures and Table

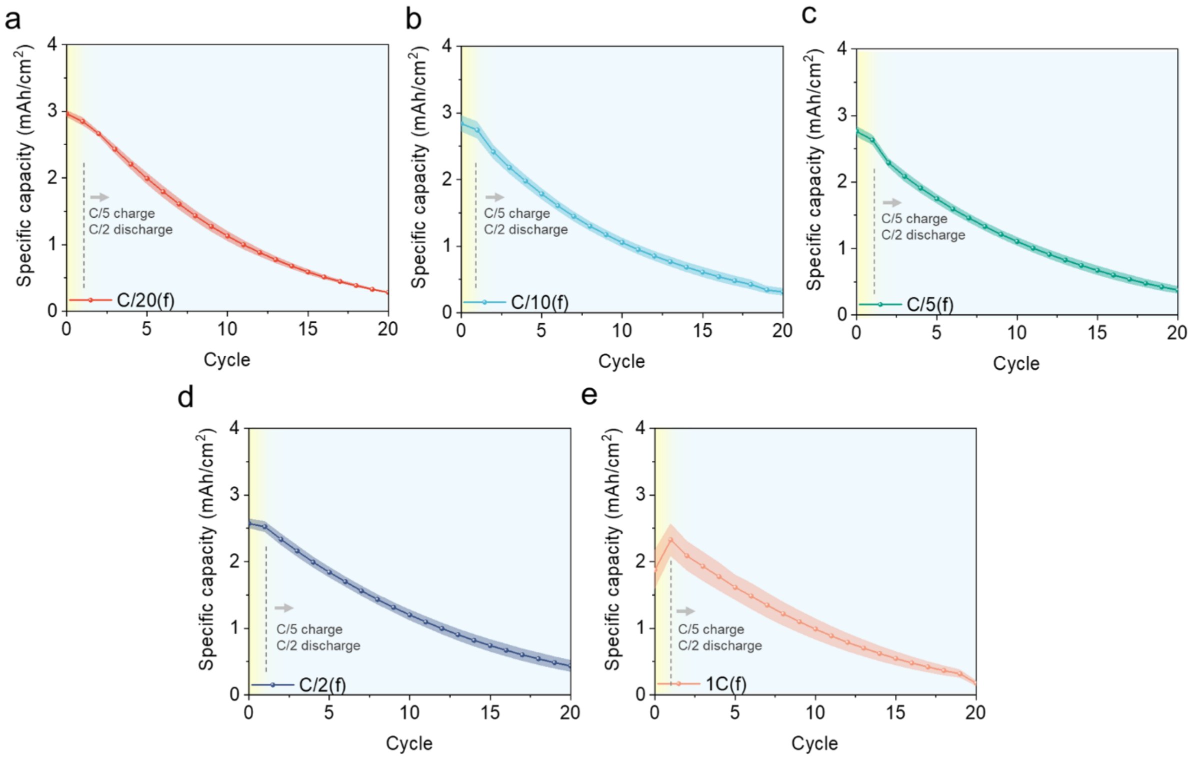

Figure S1. (a-e) Cycling performance of cells after the initial formation at different charging CDs (repeated testing for three cells)

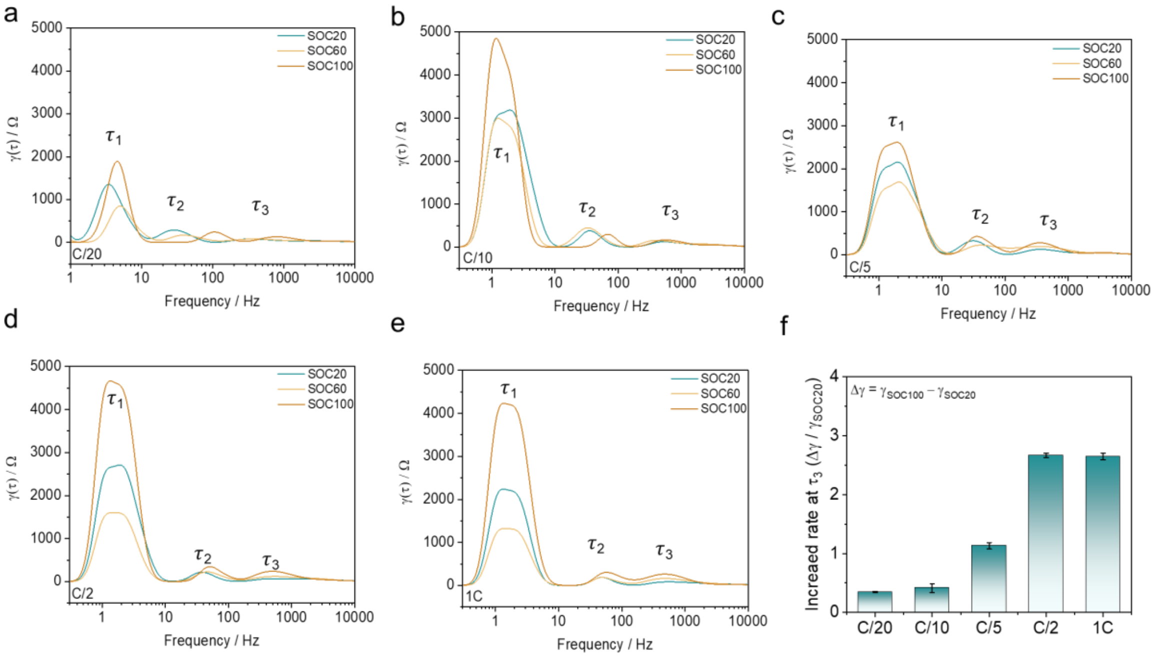

Figure S2. DRT graphs calculated from EIS measurements at state-of-charge (SoC) variations. (a) C/20 initial charging CD, (b) C/10 initial charging CD, (c) C/2 initial charging CD, (d) C/5 initial charging CD, (e) 1C initial charging CD, and (f) Increased rate of $\gamma(\tau)$ at τ_3 according to the initial charging CD.

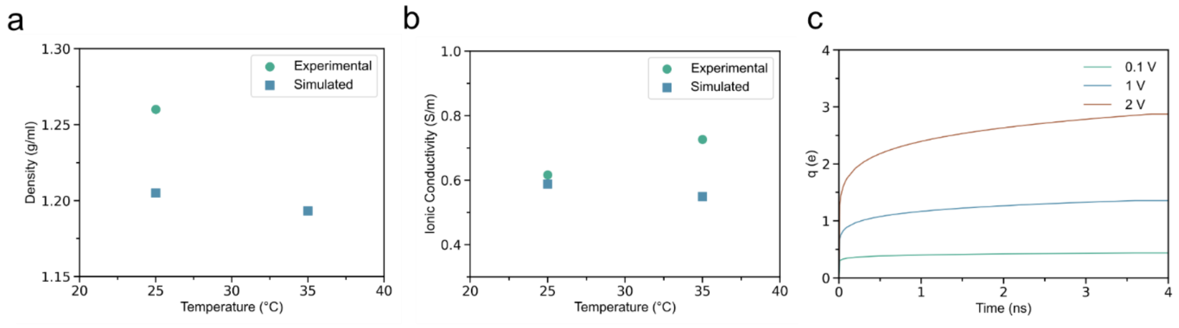

Figure S3. Molecular dynamics (MD) simulation of 1M $LiPF_6$ in EC/DEC electrolyte. (a-b) theoretical and experimental densities and ionic conductivities for the bulk system, (c) charge evolution on the Cu surface during the equilibration stage at each applied potential.

To validate the force field, we simulate 1M $LiPF_6$ in EC/DEC bulk electrolyte at 25 °C and 35 °C. We employed the Einstein–Helfand method to account for cross-interactions in the calculation of the ionic conductivity. Figure S3a-b shows the results for density and ionic conductivity. Our force field leads to an overall underprediction of these properties. The deviation in density and ionic conductivity description is 4% and 14%, respectively. These deviations agree with errors for the 0.8-scaled OPLS force field reported in the literature. [25] Moreover, the interface simulations were developed at 26 °C, where the force field showed a robust performance for experimental behavior prediction.

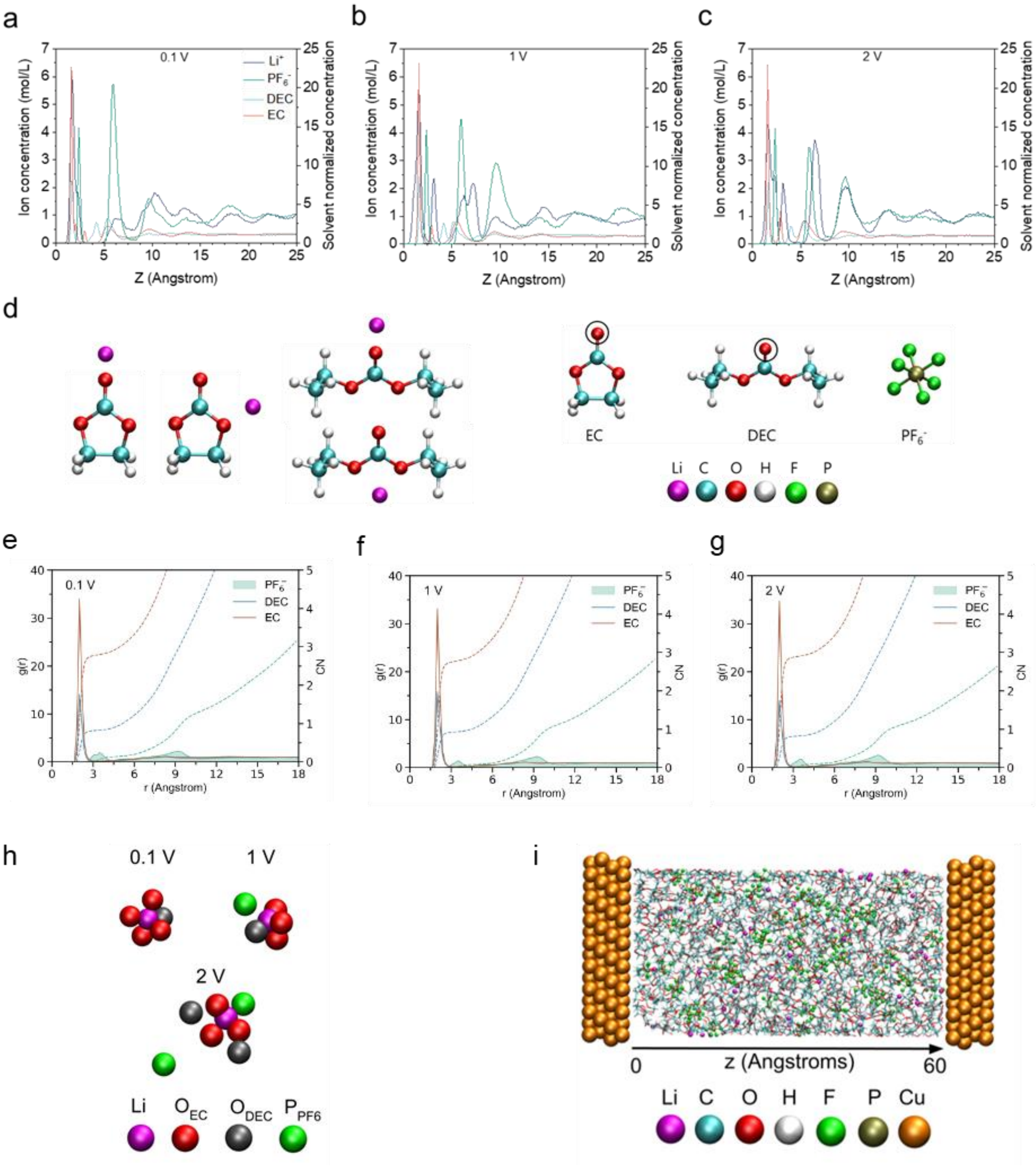

Figure S4. Molecular dynamics (MD) simulation of 1M $LiPF_6$ in EC/DEC electrolyte at Cu interface as a function of the applied potential. (a-c) local concentration profiles of ions Li^+ and PF_6^- (left y-axis) and solvents EC, and DEC (right y-axis) at the electrode interface, (d) molecular representation of Li^+ solvation modes shown in the RDFs of Figure 2 and representation of the molecules simulated, the black circle denotes the oxygen atoms from the solvents used for g(r) calculation, (e-g) the radial distribution function for Li^+-P_{PF6-}, Li^+-O_{EC}, and Li^+-O_{DEC} at the bulk layer (25-30 Å) of the simulated system, (h) the solvation shell around

Li^+ at 0.1, 1, and 2 V in the innermost layer of 5 Å, and (i) snapshot representing the simulation box composed of 1M $LiPF_6$ in EC/DEC electrolyte in contact with Cu(111) surface.

Figure S4 (e-g) presents the radial distribution function (RDF) in the bulk region of the electrolyte-Cu simulation for Li-O_{EC}, Li-O_{DEC}, and Li-P_{PF6}. The results indicate that Li^+ is primarily solvated by EC and DEC. The voltage does not significantly affect the Li^+ solvation shell. The Li^+ solvation environment in the bulk region aligns with the literature data for this class of solvents. [26]

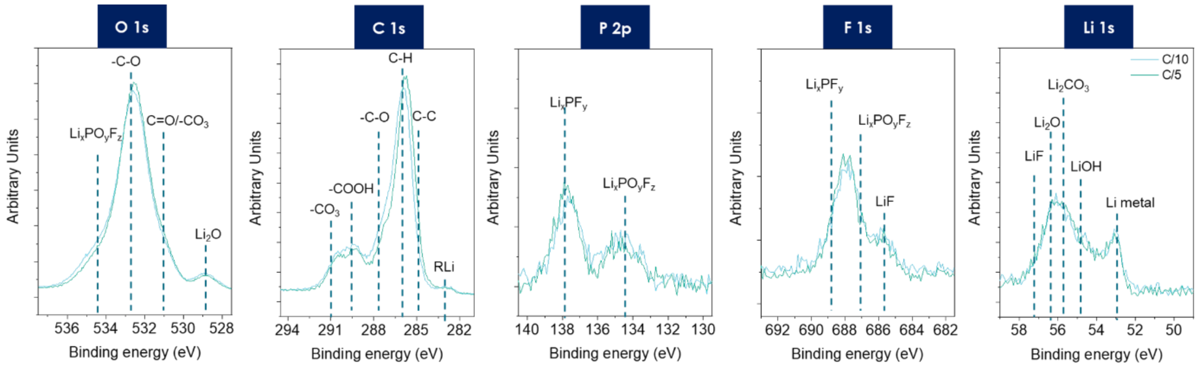

Figure S5. XPS spectra of anodes after initial charging (CD: C/10 and C/5)

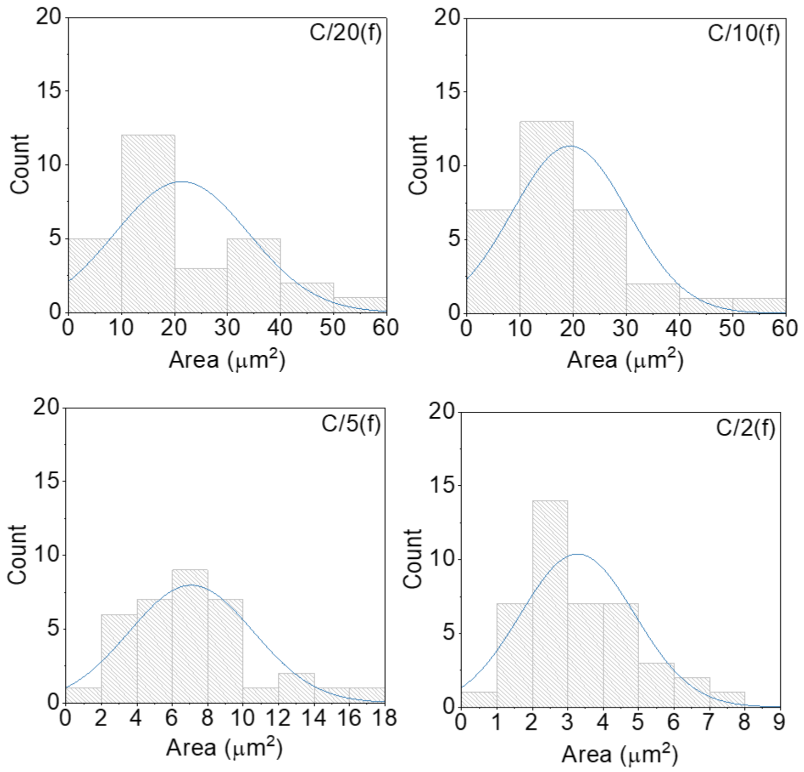

Figure S6. Calculated Li grain size (area) by image J software.

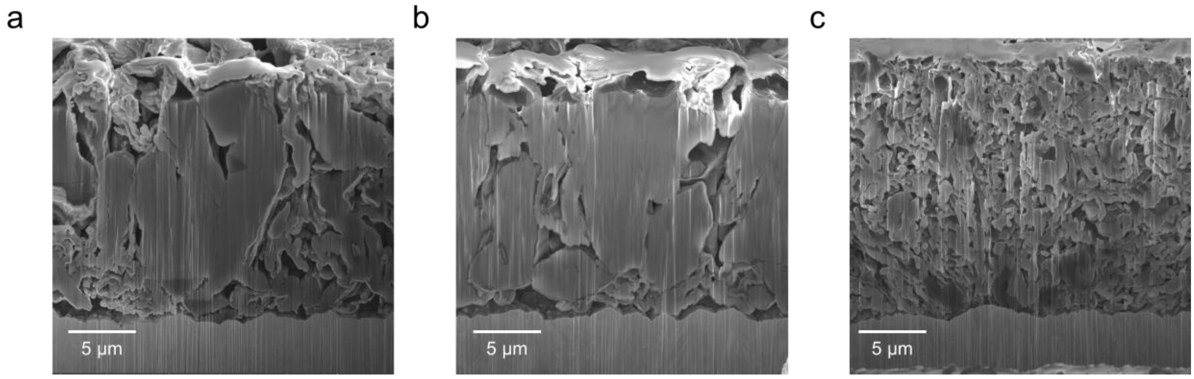

Figure S7. Cross-sectional SEM images of the 2nd charged anodes after cryo-focused ion beam milling. (a) anode with the C/20 formation cycle, (b) anode with the C/2 formation cycle, and (c) anode with the 1C formation cycle (scale bar: 5 μm)

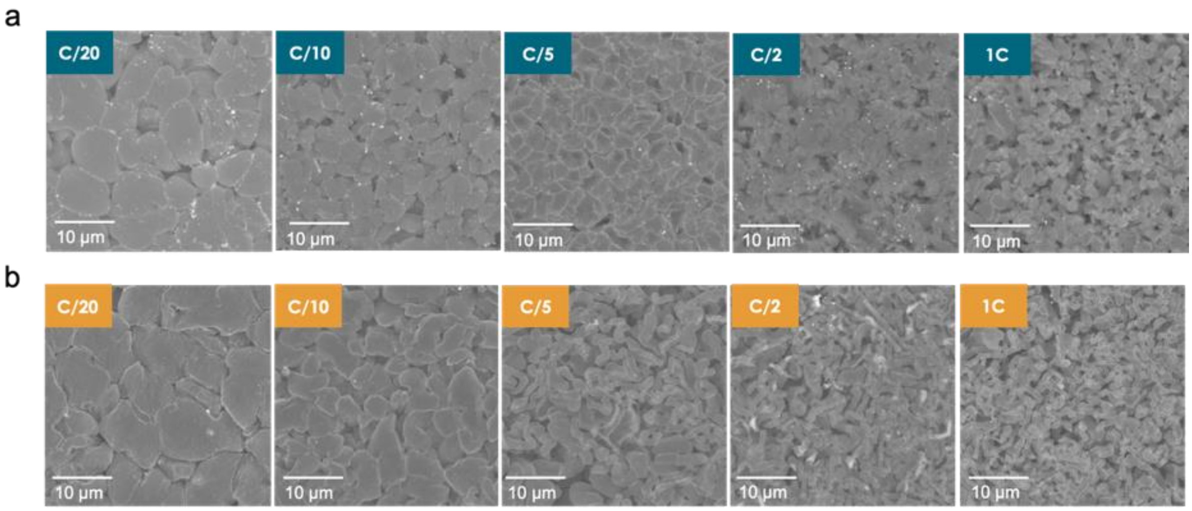

Figure S8. SEM images of anodes after initial charging. (a) with DSE and (b) with LHCE.

Table S1. Detailed Coulombic efficiency (CE) of anode-free LMBs

C/20 formation		C/10 formation		C/5 formation		C/2 formation		1C formation	
**Cycle**	**CE**	**Cycle**	**CE**	**Cycle**	**CE**	**Cycle**	**CE**	**Cycle**	**CE**
0	81.21 (±0.18)	0	78.15 (±2.04)	0	79.11 (±1.98)	0	77.42 (±1.27)	0	67.19 (±6.03)
1	100.18 (±0.17)	1	99.29 (±0.43)	1	92.25 (±0.64)	1	87.90 (±0.09)	1	83.39(±7.88)
20	85.41 (±0.62)	20	86.62 (±2.84)	20	87.86 (±0.70)	20	88.13 (±3.18)	20	84.65 (±0.48)

Table S2. Coordination Numbers obtained from the Integration of the Li^+-EC, Li^+-DEC, and Li^+-PF_6- considering the first minima in the RDF presented in Figure 2d-f.

	**0.1 V**	**2 V**
Li^+-O_{EC}	4.4	3.4
Li^+-O_{DEC}	0.5	1.2
Li^+-P_{PF6-}	0.25	0.5

3. References

(1) Louli, A. J.; Eldesoky, A.; deGooyer, J.; Coon, M.; Aiken, C. P.; Simunovic, Z.; Metzger, M.; Dahn, J. R. Different Positive Electrodes for Anode-Free Lithium Metal Cells. *J. Electrochem. Soc.* **2022**, *169* (4), 040517. DOI: 10.1149/1945-7111/ac62c4.

(2) Qian, J.; Henderson, W. A.; Xu, W.; Bhattacharya, P.; Engelhard, M.; Borodin, O.; Zhang, J.-G. High rate and stable cycling of lithium metal anode. *Nat. Commun.* **2015**, *6* (1), 6362. DOI: 10.1038/ncomms7362.

(3) Schneider, C. A.; Rasband, W. S.; Eliceiri, K. W. NIH Image to ImageJ: 25 years of image analysis. *Nat. Methods* **2012**, *9* (7), 671-675. DOI: 10.1038/nmeth.2089.

(4) Wan, T. H.; Saccoccio, M.; Chen, C.; Ciucci, F. Influence of the Discretization Methods on the Distribution of Relaxation Times Deconvolution: Implementing Radial Basis Functions with DRTtools. *Electrochim. Acta* **2015**, *184*, 483-499. DOI: https://doi.org/10.1016/j.electacta.2015.09.097.

(5) Plimpton, S. Fast Parallel Algorithms for Short-Range Molecular Dynamics. J. Comput. Phys. **1995**, *117*, 1-19. DOI: https://doi.org/10.1006/jcph.1995.1039

(6) Jorgensen, W. L; Maxwell, D. S.; Tirado-Rives, J. Development and Testing of the OPLS All-Atom Force Field on Conformational Energetics and Properties of Organic Liquids. J. Am. Chem. Soc. **1996,** *118*, 11225-11236. DOI: https://doi.org/10.1021/ja9621760

(7) Kaminski, G. A.; Friesner, R. A.; Tirado-Rives, J.; Jorgensen, W. L. Evaluation and Reparametrization of the OPLS-AA Force Field for Proteins via Comparison with Accurate Quantum Chemical Calculations on Peptides. J. Phys. Chem. B **2001**, *105*, 6474-6487. DOI: https://doi.org/10.1021/jp003919d

(8) Dodda, L. S.; de Vaca, I. C.; Tirado-Rives, J.; Jorgensen, W. L. LigParGen Web Server: an Automatic OPLS-AA Parameter Generator for Organic Ligands Nucleic Acids Res. **2017**, *45*,

W331-W336. DOI: https://doi.org/10.1093/nar/gkx312

(9) Neese, F. The ORCA Program System. WIREs Comput. Mol. Sci. **2011**, *2*, 73-78. DOI: https://doi.org/10.1002/wcms.81

(10) Lopes, J. N. C.; Padua, A. A. H. Molecular Force Field for Ionic Liquids Composed of Triflate or Bistriflylimide Anions. J. Phys. Chem. B **2004**, *108*, 16893-16898. DOI: https://doi.org/10.1021/jp0476545

(11) Jensen, K. P.; Jorgensen, W. L. Halide, Ammonium, and Alkali Metal Ion Parameters for Modeling Aqueous Solutions. J. Chem. Theory Comput. **2006**, *2*, 1499-1509. DOI: https://doi.org/10.1021/ct600252r

(12) Martínez, L.; Andrade, R.; Birgin, E. G. Martínez, J. M. PACKMOL: A Package for Building Initial Configurations for Molecular Dynamics Simulations J. Comp. Chem. **2009**, 30, 2157-2164. DOI: https://doi.org/10.1002/jcc.21224

(13) Jewett, A. I.; Stelter, D.; Lambert, J.; Saladi, S. M.; Roscioni, O. M.; Ricci, M.; Autin, L.; Maritan, M.; Bashusqeh, S. M.; Keyes, T; Dame, R. T.; Shea, J.-E.; Jensen, G. J.; Goodsell, D.S. Moltemplate: Moltemplate: A Tool for Coarse-Grained Modeling of Complex Biological Matter and Soft Condensed Matter Physics. J. Mol. Biol. 2021, 433, 166841. DOI: https://doi.org/10.1016/j.jmb.2021.166841

(14) Martyna, G. J; Tobias, D. J.; Klein, M. L. Constant Pressure Molecular Dynamics Algorithms, J. Chem. Phys. **1994**, *101*, 4177-4189. DOI: https://doi.org/10.1063/1.467468

(15) Shinoda, W.; Shiga, M.; Mikami, M. Rapid Estimation of Elastic Constants by Molecular Dynamics Simulation under Constant Stress. Phys. Rev. B: Condens. Matter Mater. Phys. **2004**, *69*, 134103. DOI: https://doi.org/10.1103/PhysRevB.69.134103

(16) Parrinello, M.; Rahman, A. Polymorphic Transitions in Single Crystals: A New Molecular Dynamics Method. J. Appl. Phys. **1981**, *52*, 7182-7190. DOI: https://doi.org/10.1063/1.328693

(17) Hockney, R. W; Eastwood, J. W. Computer Simulation Using Particles. Adam Hilger, NY, 1988.

(18) Yeh, I. C.; Berkowitz, M. L. Ewald Summation for Systems with Slab Geometry. J. Chem. Phys. **1999**, *111*, 3155-3162. DOI: https://doi.org/10.1063/1.479595

(19) Ballenegger, V.; Arnold, A.; Cerdà J. J. Simulations of Non-neutral Slab Systems with Long-range Electrostatic Interactions in Two-dimensional Periodic Boundary Conditions. J. Chem. Phys. **2009**, *131*, 094107. DOI: https://doi.org/10.1063/1.3216473

(20) Heinz, H.; Vaia, R. A.; Farmer, B. L; Naik, R. R. Simulation of Surfaces and Interfaces of fcc Metals. J. Phys. Chem. C **2008**, 112, 17281-17290. DOI: https://doi.org/10.1021/jp801931d

(21) Siepmann; J. I; Sprik, M. Influence of Surface Topology and Electrostatic Potential on Water/Electrode Systems. J. Chem. Phys. **1995**, *102*, 511-524. DOI: https://doi.org/10.1063/1.469429

(22) Reed, S. K.; Lanning, O. J.; Madden, P. A. Electrochemical Interface between an Ionic Liquid and a Model Metallic Electrode. J. Chem. Phys. **2007**, *126*, 084704. DOI: https://doi.org/10.1063/1.2464084

(23) Ahrens-Iwers, L. J. V.; Meißner, R. H. Constant Potential Simulations on a Mesh. J. Chem. Phys. **2021**, *155*, 104104. DOI: https://doi.org/10.1063/5.0063381

(24) Ahrens-Iwers, L. J. V.; Mahijs, J.; Tee, S. R.; Meißner, R. H. ELECTRODE: An electrochemistry package for LAMMPS. J. *Chem. Phys.* **2022**, 157, 084801. DOI: https://doi.org/10.1063/5.0099239

(25) Doherty, B.; Zhong, X.; Gathiaka, S.; Li, B.; Acevedo, O. Revisiting OPLS Force Field Parameters for Ionic Liquid Simulations. *J. Chem. Theory Comput.* **2017**, 13, 6131-6145. DOI: https://doi.org/10.1021/acs.jctc.7b00520

(26) Skarmoutsos, I; Ponnuchamy, V.; Vetere, V.; Mossa, S. Li+ Solvation in Pure, Binary, and Ternary Mixtures of Organic Carbonate Electrolytes. *J. Phys. Chem. C* **2015**, 119, 9, 4502-4515.DOI: 10.1021/jp511132c
